# Serum Lipids in Turkish Patients with β-Thalassemia Major and β-Thalassemia Minor

**DOI:** 10.4274/tjh.2015.0168

**Published:** 2016-02-17

**Authors:** Yasemin Işık Balcı, Şule Ünal, Fatma Gümrük

**Affiliations:** 1 Pamukkale University Faculty of Medicine, Department of Pediatric Hematology, Denizli, Turkey; 2 Hacettepe University Faculty of Medicine, Department of Pediatric Hematology, Ankara, Turkey; 3 Hacettepe University Faculty of Medicine, Department of Radiology, Ankara, Turkey

**Keywords:** Thalassemia major, Thalassemia minor, Serum lipids

## TO THE EDITOR

It is well-known that β-thalassemia is associated with changes in plasma lipids and lipoproteins [1,2,3]. To our knowledge, no data are available on lipid profiles in Turkish β-thalassemia major (TM) and β-thalassemia trait (TT) patients together. The aim of this study was to evaluate lipid profiles in two groups of patients with β-TM and β-TT and to compare them with healthy controls. The study included a total of 311 subjects. Group 1 included 131 β-TM patients (mean age: 16.3±7.58 years). Group 2 included 68 β-TT patients (mean age: 7.25±4.43 years). Group 3 consisted of 112 age- and sex-matched healthy controls (mean age: 9±4.7 years). Serum ferritin level was 2487±1103 (range: 661-5745) ng/mL in Group 1. In comparing the correlation between ferritin and lipid parameters, while a significantly negative relationship was detected between ferritin and high-density lipoprotein cholesterol (HDL-C) (p=0.000, r=-0.602), a significantly positive relationship was detected between ferritin and triglyceride (TG) levels (p=0.02) in TM patients. Serum lipid profiles of the 3 groups are shown in Table 1.

Previous studies have shown total serum cholesterol, HDL-C, lower low-density lipoprotein cholesterol (LDL-C), and higher TG in β-TM patients compared to healthy controls [1,2,3]. In our study, we found lower serum total cholesterol, lower HDL-C, LDL-C, and higher TG in β-TM patients compared to healthy controls. The pathophysiology of hypocholesterolemia in thalassemia remains obscure, although several mechanisms have been proposed; plasma dilution due to anemia, increased cholesterol requirement associated with erythroid hyperplasia, macrophage system activation with cytokine release, and increased cholesterol uptake by the reticuloendothelial system [4,5]. Previous studies reported different variations in lipid profiles of β-TT patients [6,7]. In our study, we demonstrated similar lipid profiles in β-TT patients and healthy controls. Based on statistical insignificance, we considered that the effects of lipid profile on the development of atherosclerotic vessel disease were similar in both β-TT patients and the healthy control group. Serum iron and iron stores, expressed as elevated ferritin levels, have been implicated in coronary artery disease. Iron overload depletes the antioxidant and HDL-C levels. Lower HDL-C level is an important risk factor for development of coronary heart diseases [8]. We found significant relationships of serum ferritin levels with TG and HDL-C in β-TM patients. These results indicate that β-TM patients who need life-long red blood cell transfusions should receive chelation therapy not only for iron overload-induced congestive heart failure but also in order to prevent cardiovascular diseases resulting from lipid profile alterations.

In conclusion, lipid profiles of β-TM patients differed from those of β-TT patients and healthy controls. The present study demonstrates that lower levels of HDL-C in β-TM should be a reason for concern for better evaluation of the cardiovascular risk factors in β-TM. In order to reduce the effects of lipid metabolism on cardiovascular disorders, an effective chelating therapy is essential in TM patients.

## Figures and Tables

**Table 1:**
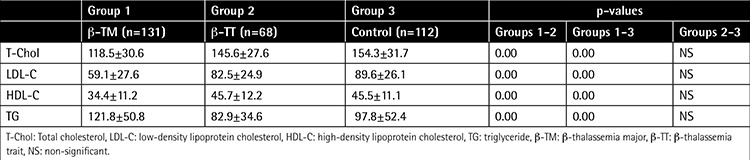
Lipid profiles and their significance in patients with β-thalassemia major, patients with β-thalassemia trait, and controls.
